# Association between Circulating Retinol Binding Protein 4, Body Mass Index, and Biomarkers of Environmental Enteric Dysfunction among Slum-Dwelling Lean Adults in Bangladesh

**DOI:** 10.4269/ajtmh.21-0322

**Published:** 2022-10-10

**Authors:** Shah Mohammad Fahim, Md. Amran Gazi, Md. Ashraful Alam, Md. Mehedi Hasan, Subhasish Das, Mustafa Mahfuz, Tahmeed Ahmed

**Affiliations:** ^1^ Nutrition and Clinical Services Division, International Centre for Diarrhoeal Disease Research, Bangladesh, Dhaka, Bangladesh;; ^2^ Faculty of Medicine and Life Sciences, University of Tampere, Finland;; ^3^ Department of Global Health, University of Washington, Seattle, Washington;; ^4^ James P Grant School of Public Health, BRAC University, Dhaka, Bangladesh

## Abstract

The relationship of retinol binding protein 4 (RBP4) with biomarkers of intestinal health and gut integrity in adults is unknown. We sought to determine the correlation between plasma RBP4 level and BMI and investigate the relationship of circulating RBP4 concentration with biomarkers of environmental enteric dysfunction among lean adults (body mass index [BMI] < 25.0 kg/m^2^) in Bangladesh. Overall, 270 adults (135 undernourished with a BMI < 18.5 kg/m^2^ and 135 healthy controls with a BMI between 18.5 and 24.9 kg/m^2^) aged 18 to 45 years were evaluated. Multivariable linear regression was performed to test the association between RBP4 and fecal biomarkers of impaired gut health. RBP4 concentration was positively correlated (rho = 0.27, *P* < 0.001) with BMI and was significantly higher in healthy controls than undernourished adults (*P* < 0.001), in male than female (*P* < 0.001), and also in employed (*P* < 0.001), smokers (*P* = 0.048) and participants with low Self-Reporting Questionnaire (SRQ)—20 scores (an instrument to screen mental health disorders) (*P* = 0.049). Statistically significant negative correlations were observed between RBP4 and fecal biomarkers of gut enteropathy including myeloperoxidase (rho = –0.23, *P* < 0.001), neopterin (rho = –0.30, *P* < 0.001), and alpha-1 anti-trypsin (rho = –0.21, *P* < 0.001). Multivariable linear regression analysis showed that increased RBP4 concentration was associated with a significant reduction in fecal neopterin (coefficient = –0.95; 95% confidence interval: –1.44 to –0.45]; *P* < 0.001) after adjustment for age, sex, nutritional status at enrollment, education, dietary diversity score, SRQ-20 score, improved sanitation, household animal exposure, and alpha-1-acid glycoprotein. The study findings revealed an inverse relationship of plasma RBP4 concentration with fecal biomarkers of altered gut health among slum-dwelling lean adults in Bangladesh.

## INTRODUCTION

Retinol binding protein 4 (RBP4), synthesized primarily in the liver, is the specific transport protein for vitamin A (retinol) in the circulation.^1^^–^^3^ RBP4 is also produced in a scanty amount by the adipose tissues and found to be positively associated with cardiometabolic risk indicators, such as elevated serum triglyceride levels, obesity, and insulin resistance.^4^^,^^5^ Circulating levels of RBP4 were measured high in individuals with type II diabetes mellitus or glucose intolerance.^4^^,^^6^ In addition, plasma levels of vitamin A bound to RBP4 were reported lower in type I diabetic patients compared with nonpatients with diabetes.^7^ A recent report from animal model studies showed that hepatocyte-derived RBP4 does not impair glucose homeostasis in mice.^8^ However, RBP4 can be affected by various factors including nutrition, vitamin A status, infections, and liver diseases.^9^ Evidence confirms that consumption of a retinoid-deficient diet can induce a loss of circulating RBP4 levels in human.^10^ Loss of RBP4 is also known to be linked with vitamin A deficiency resulting in compromised retinoid-dependent functions.^10^ It is evident in human studies that vitamin A deficiency implicates deleterious effects on the immune system of an individual and increases the susceptibility to developing infectious diseases.^11^^–^^13^ Impaired immune function predisposes to higher rates of exposure to enteric pathogens, which ultimately contributes to infiltration of inflammatory cells in the small gut lamina propria, resulting in a chronic inflammatory disorder of the small intestine.^14^^–^^16^

Environmental enteric dysfunction (EED), an asymptomatic small intestinal disorder, results in chronic intestinal inflammation and increased gut permeability. EED is rampant among the residents of unhygienic environments with the suboptimal provision of clean water supply and sanitation, resulting in frequent exposure to enteric pathogens.^17^^–^^19^ The ailment is interlinked with a complex pathway involving immune response to infections and affects the nutritional status both in children and adults.^20^^,^^21^ The implications of EED in children have been well explained,^22^ but the impact of this subclinical entity among adults living in economically disadvantaged populations remains almost unexplored. EED can be assessed by fecal biomarkers of altered gut health including myeloperoxidase (MPO), neopterin (NEO), and alpha-1 anti-trypsin (AAT).^22^^,^^23^ The concentration of MPO in stools reflects inflammatory activity in the gut mucosa, and it is considered as a potential biomarker of intestinal inflammation.^23^^,^^24^ Fecal NEO is a reliable candidate to determine small bowel mucosal damage and inflammatory changes in the gut.^25^ AAT, a marker of increased gut permeability as well as enteric loss of proteins, is considered to be an accurate diagnostic tool for assessing intestinal barrier dysfunction.^26^ The relationship between intestinal barrier function and immune response with vitamin A and RBP4 was previously investigated in children and animal models. The study documented an association between poorer vitamin A status and defective intestinal integrity among children.^27^ However, the definite role of RBP4 on body mass index (BMI) and intestinal health among adults living in resource-limited settings is unknown. We sought to determine the correlation between plasma RBP4 levels and BMI in lean adults (BMI < 25.0 kg/m^2^) living in low-income settings in Bangladesh. We also investigated whether elevated plasma RBP4 concentrations were associated with reduced expression of fecal biomarkers of EED.

## MATERIALS AND METHODS

### Study design and participants.

This study was conducted in an urban settlement in Dhaka, Bangladesh. Overall, 270 adults (135 healthy and 135 undernourished) aged between 18 and 45 years were enrolled from October 2018 to January 2019. Undernourished (BMI < 18.5 kg/m^2^) and healthy (BMI between 18.5 and 24.9 kg/m^2^) adults were defined according to the WHO criteria based on the BMI (the weight in kilograms divided by the square of the height in meters). The exclusion criteria for enrolment in the study were another family member enrolled in the same study, suffering from any severe disease requiring hospitalization, presence of congenital anomaly or chromosomal abnormalities, being pregnant or lactating woman, and presence of any known chronic illness.

### Ethics declarations.

The study protocol was approved by the Institutional Review Board of the International Center for Diarrheal Disease Research, Bangladesh (icddr,b). Informed written consent was obtained from all the participants enrolled in the study.

### Data collection.

Sociodemographic information including dietary data in the food frequency questionnaire (FFQ) and self-reporting questionnaire (SRQ-20) data were collected at the time of enrollment. FFQ is a dietary assessment tool used to identify the types and estimate the frequency of food items and beverages consumed by an individual over a specified period. SRQ-20 is an instrument used to screen mental health disorders and detect psychological symptoms in adults.^28^ BMI was calculated from the height and weight of the participants measured by the trained field staff at enrolment before biological sample collection.

### Sample collection and storage.

Two milliliters of blood samples were collected aseptically into blood collection tubes (S-Monovette 7.5 mL, Sarstedt) by venipuncture from all the participants. To separate the plasma, blood samples were centrifuged at 4,000 × *g* for 10 minutes within 2 hours of collection. Stool samples were collected and preserved in a liquid nitrogen tank within 20 minutes of production. No additive or preservative was added to the fecal samples. All the samples were stored at –80°C, until analysis.

### Laboratory analysis.

Laboratory assays were performed in the parasitology laboratory at icddr,b in Dhaka, Bangladesh. The biomarkers were tested in a single batch to minimize potential intra- and interbatch variations. Plasma RBP4 (R&D Systems Inc, Minneapolis, MN) and blood biomarkers such as C-reactive protein (CRP; Immundiagnostik, Bensheim, Germany), alpha 1-acid glycoprotein (AGP) (Alpco, Salem, NH, USA), low-density lipoprotein receptor-related protein 1 (LRP1; Biomatik, Wilmington, DE) and Ferritin (ORGENTEC Diagnostika GmbH, Mainz, Germany) were estimated using commercial ELISA kits following the instructions provided by the manufacturers. Hemoglobin status was determined using Hb 201 microcuvette of HemoCue Company. Plasma zinc was measured by the atomic absorption spectrometry method. Stool biomarkers were measured through ELISA according to the standard protocols given by the manufacturers. Stool samples were analyzed for MPO (Alpco, Salem, NH), NEO (GenWay Biotech, San Diego, CA), and AAT (Biovendor, Chandler, NC). *Helicobacter pylori* fecal antigen test was also performed using Amplified IDEIA Hp StAR (OXOID Limited, Hampshire, UK).

### Variables measured in this analysis.

The following variables were included in the study: demographic (age, sex, education, employment status); socioeconomic (family income, household animal exposure); water, sanitation, and hygiene practices (hand washes before taking or preparing foods and after using the toilet, improved water sources, improved sanitation, use of toilet paper); personal (SRQ-20 score, smoking, substance use); dietary (dietary diversity score calculated from FFQ); markers of systemic inflammation (CRP, AGP); biochemical (hemoglobin, ferritin, zinc); fecal biomarkers of altered gut health (MPO, NEO, AAT); enteric infection (*H. pylori* infection); plasma LRP1; plasma RBP4; and anthropometric variable (BMI). Dietary diversity score and SRQ-20 score were categorized based on previously published literature.^29^^–^^31^

### Statistical analysis.

Results are expressed as mean (± SD) for normally distributed continuous variables and median with interquartile range (IQR) for asymmetric numeric variables. A proportion estimate was used to summarize the categorical variables. Nonparametric Mann–Whitney test was performed to examine the differences in RBP4 levels between the comparison groups. Spearman’s correlation coefficient (rho) was calculated to analyze the correlations of RBP4 with fecal biomarkers of altered gut health, inflammatory biomarkers, and plasma micronutrient levels of the participants. Multivariable linear regression was performed to test the association between RBP4 and fecal biomarkers of altered intestinal health after controlling for the potential confounders. We constructed three independent linear regression models considering RBP4 as the explanatory variable and MPO, NEO, and AAT as the outcome variables, respectively. Variables with skewed distribution (RBP4, MPO, NEO, AAT, AGP, CRP, and LRP1) were log-transformed before regression analyses. Covariates were included in each multivariable model if the *P* value was found to be < 0.2 in the univariate analysis. Multicollinearity was estimated by variance inflation factors (VIF). However, we did not exclude any variables for multicollinearity as VIF values were found to be < 4.0 in all the regression models. A two-tailed *P* value < 0.05 was considered statistically significant. All the analyses were performed using R version 3.5.3 (https://www.r-project.org, Foundation for Statistical Computing, Vienna, Austria) software.

## RESULTS

### General characteristics of the study population.

A total of 270 participants with a mean (± SD) age of 24.2 (± 6.6) years were enrolled in this study. Of them, 95 (35.2%) were used, and 24 (8.9%) were smokers. The sample comprised 205 (75.9%) females, with 110 (53.7%) assigned to the undernourished group and 95 (46.3%) to the healthy group. The mean (± SD) BMI of the participants was 19.6 (± 2.6). The median value with IQR of hemoglobin was 12.8 (11.7–13.8) gm/dL, ferritin was 50.6 (22.2–99.7) ng/mL, and plasma zinc was 0.73 (0.66–0.82) mg/L. The distribution of sociodemographic, biochemical, and outcome characteristics of the participants are shown in Table 1.

**Table 1:** Sociodemographic, biochemical, and outcome characteristics of the participants*

Variables	Undernourished (*N* = 135)	Healthy (*N* = 135)	Total (*N* = 270)
Age (years), m (SD)	23.4 (± 6.6)	25.1 (± 6.6)	24.2 (± 6.6)
Sex (female), *n* (%)	110 (81.5%)	95 (70.4%)	205 (75.9%)
BMI, kg/m^2^	17.3 (± 0.7)	21.8 (± 1.8)	19.6 (± 2.6)
Family income, USD†	177.9 (118.6–237.3)	213.5 (154.2–296.6)	177.9 (142.4–261.0)
Education (received), *n* (%)	118 (87.4%)	118 (87.4%)	236 (87.4%)
Employment (yes), *n* (%)	40 (29.6%)	55 (40.7%)	95 (35.2%)
Dietary diversity score (high), *n* (%)	44 (32.6%)	62 (45.9%)	106 (39.3%)
SRQ-20 score (high), *n* (%)	69 (51.1%)	40 (29.6%)	109 (40.4%)
Smoking (yes), *n* (%)	6 (4.4%)	18 (13.3%)	24 (8.9%)
Substance use (yes), *n* (%)	9 (6.7%)	9 (6.7%)	18 (6.7%)
Improved water source (yes), *n* (%)	135 (100%)	135 (100%)	270 (100%)
Improved sanitation (yes), *n* (%)	29 (21.5%)	28 (20.7%)	57 (21.1%)
Use of toilet paper (yes), *n* (%)	25 (18.5%)	44 (32.6%)	69 (25.6%)
Household animal exposure (yes), *n* (%)	6 (4.4%)	9 (6.7%)	15 (5.9%)
Hemoglobin, gm/dL	12.4 (11.5–13.4)	13.2 (12.0–14.3)	12.8 (11.7–13.8)
Ferritin, ng/mL	43.7 (20.2–80.1)	59.9 (27.3–122.7)	50.6 (22.2–99.7)
Zinc, mg/L	0.71 (0.63–0.81)	0.74 (0.68–0.84)	0.73 (0.66–0.82)
CRP, mg/L	0.8 (0.3–1.8)	2.1 (0.9–4.4)	1.4 (0.5–3.4)
AGP, mg/dL	64.7 (52.9–87.0)	75.8 (63.0–95.5)	71.0 (57.0–88.9)
LRP1, ng/mL	707.8 (590.7–839.1)	1673.2 (1386.6–1885.0)	1044.0 (700.0–1673.2)
MPO, ng/mL	388.5 (256.5–597.0)	104.5 (59.0–202.0)	245.8 (100.3–541.5)
NEO, nmol/L	408.0 (242.0–730.0)	353.0 (182.5–651.0)	390.5 (210–667.5)
AAT, mg/g	0.19 (0.08–0.40)	0.12 (0.05–0.23)	0.16 (0.06–0.32)

AGP = alpha 1-acid glycoprotein; CRP = C-reactive protein; LRP1: low-density lipoprotein receptor-related protein 1; SRQ-20 = Self-Reporting Questionnaire—20.

* Descriptive characteristics are presented in n (%), Mean (±SD) or Median (IQR), as appropriate.

† At the time of writing, 1 U.S. dollar (USD) was equivalent to 84.3 Bangladeshi taka.

### RBP4 concentration in the study population and correlation with BMI.

**Figure 1.**
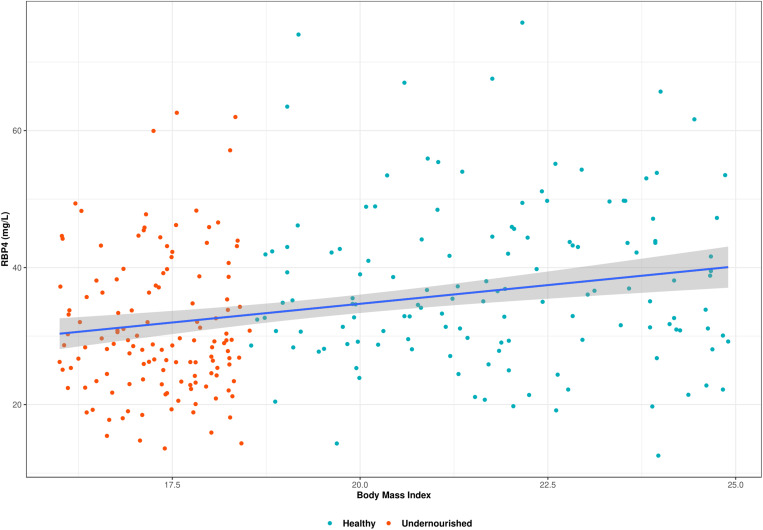
The correlation between retinol binding protein 4 (RBP4) and body mass index is shown in a scatter plot with regression line. It shows a significant positive correlation between RBP4 and body mass index (rho = 0.27, *P* < 0.001).

Figure 1 presents a scatter plot of correlation between RBP4 and BMI of the study participants. Plasma RBP4 concentration was positively correlated with BMI (rho = 0.27, *P* < 0.001). We also observed that RBP4 concentration was higher (median = 35.1 mg/L, IQR = 29.4–43.8) in the healthy controls than in the undernourished adults (median = 28.8 mg/L, IQR = 23.8–37.2) and the difference was statistically significant (*P* < 0.001).

### Distribution of RBP4 in different comparison groups.

Table 2 describes the differences in RBP4 concentration among different comparison groups. Circulating RBP4 concentration was significantly higher in males than in the female participants (*P* < 0.001). RBP4 levels were increased in employed (*P* < 0.001), smokers (*P* = 0.048), and participants with low SRQ-20 scores (*P* = 0.049). There were no significant differences in plasma RBP4 concentration between the groups based on education, dietary diversity score, substance abuse, and *H. pylori* infection.

**Table 2:** Differences in RBP4 concentration by sociodemographic variables and micronutrient status*

		RBP4, mg/L	
Variables	*n*	Median (IQR, Q1–Q3)	*P* value
Sex			
Male	65	35.6 (29.7–43.3)	< 0.001
Female	205	30.7 (24.6–39.8)	
Education			
Received	236	31.7 (26.4–42.3)	0.85
Not received	34	33.3 (26.2–38.7)	
Employment			
Yes	95	35.3 (29.1–43.8)	< 0.001
No	175	30.3 (24.5–38.7)	
Dietary diversity score			
High (score > 4)	106	32.8 (27.8–40.7)	0.46
Low (score ≤ 4)	164	31.2 (26.1–43.0)	
SRQ-20 score			
High (score ≥ 7)	109	30.3 (24.3–39.3)	0.049
Low (score < 7)	161	32.8 (28.1–43.0)	
Smoking			
Yes	24	35.8 (29.5–42.1)	0.048
No	246	31.3 (25.9–41.9)	
Substance use			
Yes	18	32.7 (27.9–38.1)	0.97
No	251	31.6 (26.2–42.1)	
*Helicobacter pylori* infection			
Yes	151	32.7 (26.2–41.9)	0.77
No	117	31.2 (26.4–42.0)	

SRQ-20 = Self-Reporting Questionnaire—20.

* Mann-Whitney *U* tests were applied to test differences between the groups.

### Correlation of RBP4 with different biomarkers and plasma micronutrient levels.

**Figure 2.**
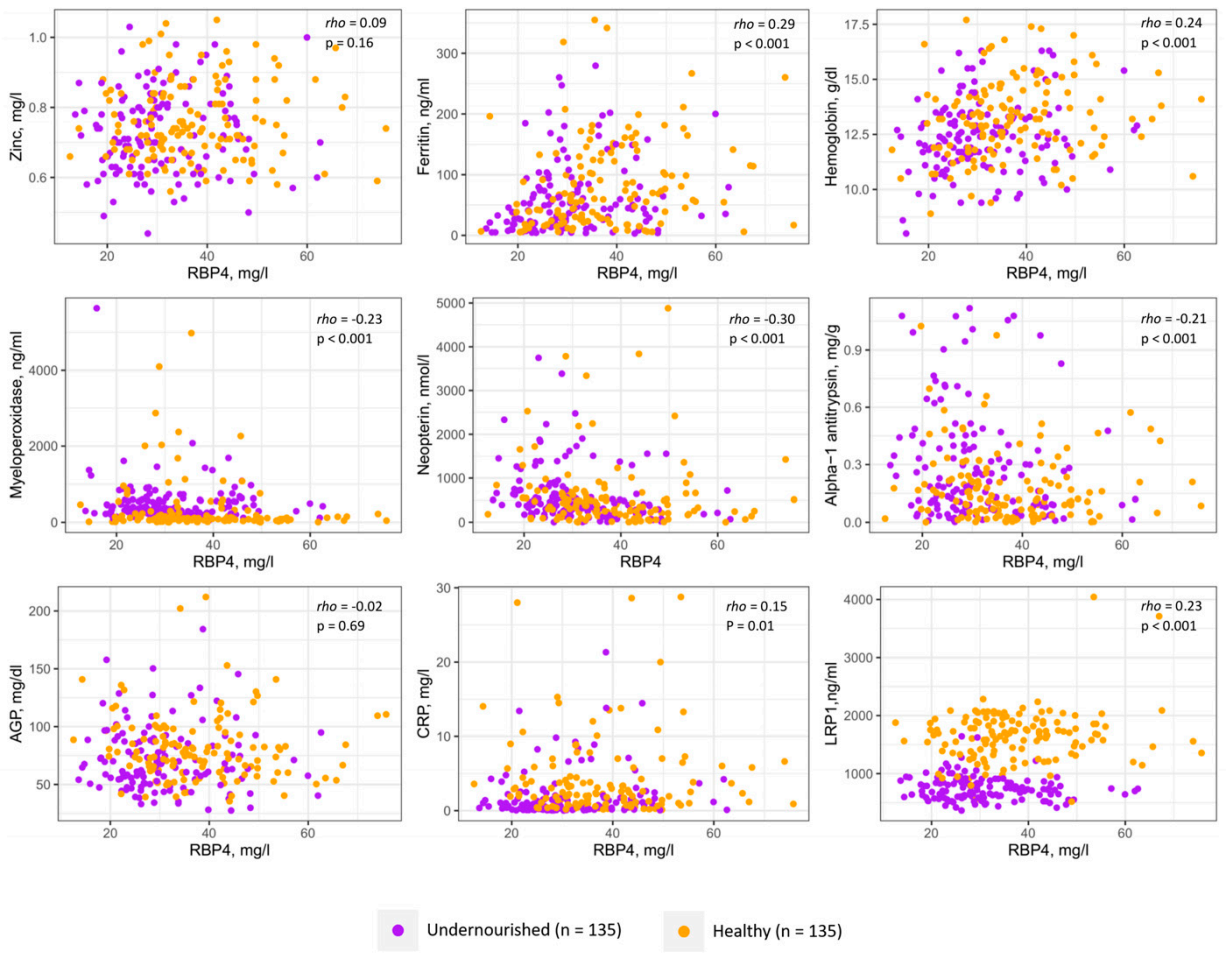
Multipanel scatter plots showing the correlations of retinol binding protein 4 (RBP4) with biomarkers of altered gut health, systemic inflammation, and micronutrient status of the adult participants both in healthy and undernourished groups. rho represents the spearman’s correlation coefficient between RBP4 and the individual biomarker, and *P* values indicate statistical significance. AGP = alpha-1-acid glycoprotein; CRP = C-reactive protein; LRP1 = low-density lipoprotein receptor-related protein 1.

Figure 2 exhibits results of the Spearman’s correlation test between RBP4 and biomarkers of altered gut health, systemic inflammation, and micronutrient status. We observed statistically significant negative correlations between RBP4 and the fecal biomarkers of EED including myeloperoxidase (rho = –0.23, *P* < 0.001), neopterin (rho = –0.30, *P* < 0.001), and alpha-1 anti-trypsin (rho = –0.21, *P* < 0.001). RBP4 was positively correlated with ferritin (rho = 0.29, *P* < 0.001), hemoglobin (rho = 0.24, *P* < 0.001) and LRP1 (rho = 0.23, *P* < 0.001) concentrations measured in plasma. A significant positive correlation was also observed between RBP4 and CRP (rho = 0.15, *P* = 0.01) in adults enrolled in this study.

### Association of RBP4 with fecal biomarkers of altered gut health.

Table 3 displays results of the adjusted linear regression analyses on the relationship between RBP4 and fecal biomarkers of EED. Increased RBP4 concentration was associated with a significant reduction in fecal NEO (coefficient = –0.95; 95% confidence interval: –1.44 to –0.45; *P* < 0.001) after adjustment for age, sex, nutritional status at enrollment, education, dietary diversity score, SRQ-20 score, improved sanitation, household animal exposure, and AGP as confounders. However, the relationship of circulating RBP4 with fecal MPO and AAT did not demonstrate any statistically significant relationship in multivariable linear regression analyses.

**Table 3:** Association of RBP4 with fecal biomarkers of altered gut health using multivariable linear regression analyses*†

	MPO (ng/mL)‡		NEO (nmol/L)§		AAT (mg/g)‖	
	Coefficient (SE)	*P* value	Coefficient (SE)	*P* value	Coefficient (SE)	*P* value
RBP4	−0.22 (0.23)	0.33	−0.95 (0.25)	<0.001	−0.42 (0.27)	0.12
Age in years	−0.01 (0.01)	0.37	−0.01 (0.01)	0.88	0.01 (0.01)	0.99
Sex (female)	−0.21 (0.22)	0.34	−0.31 (0.19)	0.10	−0.20 (0.24)	0.41
Nutritional status at enrollment (Undernourished)	1.24 (0.25)	<0.001	0.23 (0.17)	0.18	−0.07 (0.31)	0.82
Education received (yes)	NS¶	NS	0.54 (0.26)	0.04	NS	NS
Employment (yes)	−0.33 (0.17)	0.05	NS	NS	−0.29 (0.21)	0.16
Dietary diversity score	NS	NS	0.09 (0.06)	0.15	NS	NS
SRQ-20 score	NS	NS	0.02 (0.02)	0.18	NS	NS
Always wash hands before taking or preparing a meal (yes)	NS	NS	NS	NS	−0.36 (0.21)	0.09
Improved sanitation (yes)	NS	NS	0.03 (0.20)	0.86	NS	NS
Use of toilet paper (yes)	−0.12 (0.16)	0.47	NS	NS	NS	NS
Household animal exposure (yes)	NS	NS	0.34 (0.34)	0.31	NS	NS
Hemoglobin	0.07 (0.66)	0.91	NS	NS	NS	NS
Ferritin	−0.06 (0.08)	0.41	NS	NS	NS	NS
Zinc	−0.28 (0.48)	0.56	NS	NS	NS	NS
AGP	−0.28 (0.21)	0.18	0.70 (0.23)	<0.001	NS	NS
CRP	0.14 (0.05)	0.01	NS	NS	−0.01 (0.06)	0.83
LRP1	0.06 (0.25)	0.82	NS	NS	−0.58 (0.31)	0.06

AGP = alpha 1-acid glycoprotein; CRP = C-reactive protein; LRP1: low-density lipoprotein receptor-related protein 1; RBP4 = retinol binding protein 4; SRQ-20 = Self-Reporting Questionnaire—20.

* Myeloperoxidase, neopterin, and alpha-1 anti-trypsin were the outcomes of three independent linear regression models, and the results of multivariable analyses reported in the table.

† RBP4, Myeloperoxidase, neopterin, and alpha-1 anti-trypsin, AGP, CRP, and LRP1 were log-transformed before regression analysis.

‡ Adjusted for age, sex, nutritional status at enrollment, employment status, use of toilet paper, hemoglobin, ferritin, zinc, AGP, CRP, and LRP1.

§ Adjusted for age, sex, nutritional status at enrollment, education received or not, dietary diversity score, SRQ-20 score, improved sanitation, household animal exposure, and AGP.

‖ Adjusted for age, sex, nutritional status at enrollment, employment status, always washing hands before taking or preparing a meal, CRP, and LRP1.

¶ NS denotes to the variables with a *P* value ≥ 0.2 on univariate analyses and thus not included in the multivariable models.

## DISCUSSION

In this study involving 270 young adults, we observed that plasma RBP4 concentration was significantly lower in underweight adults than in the healthy controls, and BMI was positively correlated with RBP4 levels in the bloodstream. The fecal biomarker values measured in the stool samples were higher in undernourished participants than in healthy adults and exhibited negative correlations with RBP4. The study findings also demonstrated a significant inverse relationship between RBP4 and fecal biomarker of intestinal inflammation. Because of the lack of evidence on the relationship between biomarkers of EED and adipokines, the findings from this study will provide further insights to understand the complex mechanism of EED in young adults.

Our finding on the positive correlation between RBP4 and BMI is consistent with previous studies done on adult participants.^32^^–^^35^ Earlier evidence showed that RBP4 levels were lower in ill patients compared with healthy or relatively stable participants, indicating that RBP4 can be downregulated under stressful conditions, such as nutritional impairment.^36^^–^^38^ A study done on children with protein energy malnutrition reported a lower level of RBP4 among the study participants, which improved after increased energy intake and protein therapy.^39^ RBP4, the transporter of vitamin A to peripheral tissues through circulation, is considered to be a proxy indicator of vitamin A and plays a potential role in apoptosis as well as cellular proliferation and differentiation.^32^^,^^40^ A study conducted on nonobese healthy Spanish women revealed a positive association between vitamin A intake and RBP4 concentrations.^41^ To that end, it is possible that vitamin A metabolism might be dysregulated in underweight adults; therefore, a reduced expression of RBP4 is observed in their circulation. Moreover, RBP4 is a negative-acute phase reactant, and it can be reduced during malnutrition and inflammatory conditions, even in subclinical infections.^42^ Because the undernourished adults enrolled in this study are dwellers of an unhygienic environment, they are more likely to be exposed to multiple enteric pathogens, resulting in persistent insults to their small gut as well as subclinical enteric infections. This can be a potential reason for having lower concentrations of RBP4 among the underweight adults of this study. Perhaps, human body induces an acute phase response during undernutrition that is attributable to the downregulation of negative acute-phase proteins such as RBP4. Additionally, RBP4 can modify the function of transthyretin.^43^ Transthyretin is the transport protein for thyroxine and is considered a marker for nutritional status assessment.^38^^,^^44^ The metabolic pathways involving RBP4 and transthyretin may also be implicated in the reduction of RBP4 in underweight adults.^45^

The average fecal biomarker values were markedly raised in our study participants against nontropical normal values (MPO < 2000 ng/mL, NEO < 70 nmol/L, and AAT < 0.27 mg/g).^26^ This study was restricted to adults, and the findings of elevated fecal biomarker values suggest that adults enrolled in this study are living in an environment exposed to constant sources of chronic inflammation. Our results demonstrated that small intestinal lesion is highly persistent in Bangladeshi adults, even in apparently healthy subjects with no symptoms of disease. In this study, circulating RBP4 concentration was negatively associated with fecal neopterin supporting that RBP4, a negative acute-phase protein, can be reduced during subclinical infections or in inflammatory conditions.^46^^–^^48^ Because RBP4 is known for the transport of vitamin A to the peripheral tissues, a vitamin A–dependent mechanism underlying its effect on small intestinal enteropathy seems to be a reasonable assumption. Apart from its role in vision, vitamin A is responsible for the maintenance of epithelial integrity and immune functions in adults.^49^^,^^50^ Therefore, the negative correlation of circulating RBP4 levels with myeloperoxidase and neopterin suggests that the expression of RBP4 reduced markedly in adults with probable chronic gut inflammation owing to loss of integrity of the epithelial surface in the small intestine.^51^ Nevertheless, vitamin A is an antiinflammatory agent with immunomodulatory effects, and this explains our finding on the inverse relationship between RBP4 and fecal neopterin, an indicator of cellular immune activation as well as intestinal inflammation.^23^^,^^51^^–^^53^ However, we studied lean adults, and all were from slum areas; additional studies are needed in more diverse groups to ascertain the findings.

Evidence suggests that the rise of positive acute-phase proteins with a simultaneous decrease in negative acute phase proteins or visceral proteins indicate the presence of inflammatory process within the body.^54^^–^^56^ Therefore, the inverse correlation between plasma RBP4 levels and fecal AAT, a positive acute phase protein, suggests the existence of an inflammatory process in the small gut of the adults enrolled in this study. RBP4 is considered a marker of subclinical protein-calorie malnutrition, which is found to be lower in patients with inflammatory bowel disease (IBD).^57^^,^^58^ Fecal AAT concentrations were estimated to be higher in patients with IBD, and elevated fecal AAT concentrations were negatively correlated with RBP4.^57^ A study on women with epithelial ovarian cancer reported decreased levels of serum RBP4 among the patients with a simultaneous increase in AAT concentrations.^59^ Consistent with those studies, our finding on the inverse relationship between AAT and RBP4 ostensibly appears to point to the conclusion that there can be increased gut permeability as well as intestinal protein loss in adults with low RBP4 levels in the circulation. This finding has implications for understanding the contribution of suboptimal RBP4 levels in the pathogenesis of EED. It will also provide rationale to investigate the role of vitamin A as an antiinflammatory mediator to ameliorate the characteristic features of EED.

Our study has several strengths and also potential shortcomings. To the best of our knowledge, this is the first study to demonstrate the relationship between plasma RBP4 concentration and biomarkers of EED in adults living in low-income settings. The use of multiple measures of EED is a strength of this analysis. We used multiple procedures to ensure compliance and reliability of data, sample, and analysis according to standard operating protocols. Additionally, several possible confounding variables were taken into consideration in this analysis that are possible risk factors for altered gut health in adults, including age, sex, education, occupation, income, dietary diversity, water, sanitation and hygiene practice, and smoking history. Limitations of our study include a lack of data regarding body fat percentage, lipid variables, fasting blood glucose, physical activity, transthyretin, insulin concentration, blood pressure, and dietary intakes of short chain fatty acids and triglycerides. These variables should be adjusted to determine the precise relationship between RBP4 and biomarkers of altered gut health. Moreover, RBP4 concentration in plasma depends on the vitamin A status of an individual.^46^ Hence, serum retinol and dietary intake of vitamin A should be included in the regression model as covariates. Circulating RBP4 level is more linked to visceral fat than subcutaneous fat. Therefore, the inclusion of waist circumference and waist-to-hip ratio as potential covariates should be considered. Moreover, the lack of ability to imply causality and the presence of bias associated with the cross-sectional nature of the study can also be raised for this analysis.

In conclusion, RBP4 was positively correlated with the BMI of slum-dwelling lean adults in Bangladesh. Our findings also revealed the inverse relationship of plasma RBP4 levels with fecal biomarkers of altered gut health, suggesting that biological mechanisms influencing RPB4 concentration may be related to the impairment of gut function. Further research, including well-designed longitudinal studies considering larger sample sizes and all possible confounders, is needed to confirm the potential effects of RBP4 on intestinal health and clarify the role of impaired vitamin A metabolism in the pathogenesis of altered gut function as well as EED in malnourished adults living in resource-poor settings.
